# Development and internal validation of a prediction model for clinical pregnancy in GnRH antagonist cycles: a retrospective cohort study

**DOI:** 10.3389/fendo.2026.1916298

**Published:** 2026-07-28

**Authors:** Jin Shang, Ying Huang, Dan Jin, Wenjing Zhou, Jing Yang, Ling Zhou, Lanmei Zhang, Dongdong Ni

**Affiliations:** 1 Reproductive Medical Center, The Ninth Medical Center of PLA General Hospital, Beijing, China; 2 Gynaecology and Obstetrics, The Ninth Medical Center of PLA General Hospital, Beijing, China

**Keywords:** assisted reproductive technology, Boruta algorithm, GnRH antagonist protocol, LASSO regression, luteinizing hormone alterations, pregnancy outcomes

## Abstract

**Background:**

In clinical practice, the management of luteinizing hormone (LH) levels during controlled ovarian hyperstimulation (COH) with gonadotropin releasing hormone antagonist (GnRH-ant) protocols presents a significant challenge that can influence *in-vitro* fertilization (IVF) outcomes. This complex issue requires comprehensive consideration of multiple interrelated factors, including patient age, ovarian response, and other hormonal parameters. Currently, no consensus has been established regarding the optimal approach to integrating these variables and determining their weights to achieve the most favorable pregnancy outcomes.

**Objective:**

This study aimed to identify key determinants of IVF outcomes and to develop predictive models for transferable embryo yield, cumulative pregnancy, and live birth in assisted reproductive technology (ART).

**Study design:**

This retrospective cohort study enrolled 570 patients who underwent the GnRH-ant protocol between January 2020 and January 2025. All eligible patients were randomly divided into a training set and a validation set. The Boruta algorithm and LASSO regression were applied to identify key clinical predictors for the number of transferable embryos, cumulative pregnancy, and live birth, respectively. Because the predictor set associated with pregnancy showed the greatest overlap with those for available embryo and delivery, a multivariable logistic regression model was built with pregnancy as the primary outcome, and a nomogram was constructed. The model’s discrimination and calibration were assessed in the training set and evaluated in an internal split-sample validation set.

**Results:**

A total of 570 patients were included in the analysis. Among 21 candidate variables, six features-age, antral follicle count (AFC), the baseline follicle-stimulating hormone (FSH) and LH on the 2nd or 3th day of menstruation, estradiol (E2) level on the day of human chorionic gonadotropin (HCG) administration and LH alterations—were consistently identified as significant predictors. A nomogram incorporating these factors was developed. The model yielded AUCs of 0.715 (95% CI, 0.658-0.771) in the training set and 0.662 (95% CI, 0.565-0.759) in the validation cohort. Calibration curves demonstrated agreement between predicted and observed clinical pregnancy probabilities.

**Conclusion:**

LH alterations during controlled ovarian hyperstimulation was associated with clinical outcomes in GnRH antagonist cycles, and may serve as a candidate dynamic marker for further investigation.

## Introduction

In assisted reproductive technology (ART), gonadotropin-releasing hormone antagonist (GnRH-ant) have long been central to controlled ovarian hyperstimulation (COH) protocols (1). By inducing pituitary down-regulation, GnRH-ant effectively suppress premature luteinizing hormone (LH) surges, thereby enhancing follicular developmental synchrony (2). More recently, GnRH-ant protocols have gained considerable clinical traction, owing to a more favorable physiological profile that includes shorter stimulation duration, reduced treatment costs, and a lower incidence of ovarian hyperstimulation syndrome (OHSS) (3).

At physiological concentrations, LH exerts its biological effects on oocyte development by inducing extreme reorganization in follicular cells, which in turn results in ovulation and luteinization. A premature LH surge can trigger untimely ovulation and follicular luteinization, adversely affecting oocyte retrieval rates, embryo quality, fertilization rates, implantation potential, and clinical pregnancy outcomes (4). Insufficient LH levels during the follicular phase can impair estrogen synthesis and secretion, adversely affecting both follicular development and endometrial receptivity (5). During *in-vitro* fertilization (IVF), the GnRH-ant protocol causes profound suppression of endogenous LH, which may negatively impact follicular development. The normal physiological function of LH requires a certain concentration range, namely, the “LH window”, according to some studies, the optimal LH window ranges from 1.2 to 5 IU/L (6, 7). However, some reports indicate that even with LH levels <1.2 IU/L in COH, pregnancy outcomes remain unaffected (8). In clinical practice, when using antagonist protocols for COH, we have observed that lower LH levels on the day of Human Chorionic Gonadotropin (HCG) administration may result in fewer high-quality embryos than expected in some patients with normal ovarian function consistent with the conclusions of other studies (9), and we found that patients who significantly reduced their LH levels through antagonists tended to have poor pregnancy outcomes.

Previous studies have predominantly established a unilinear correlation between LH level fluctuations and assisted reproductive outcomes, while a comprehensive predictive framework accounting for the collective influence of other factors on pregnancy outcome remains lacking. In this retrospective study, we evaluated the predictive value of serum LH levels and other factors at two key time points-baseline (cycle day 2-3) and the day of HCG trigger-during gonadotropin-releasing GnRH-ant controlled ovarian stimulation (COS). The objective was to assess their utility in forecasting ovarian response and subsequent reproductive outcomes. These findings may assist in individualizing GnRH-ant protocols and optimizing COS management to improve clinical results.

## Materials and methods

### Study design and participants

This retrospective cohort study was performed at the Ninth medical center of Chinese PLA General Hospital from January 2020 to January 2025. The study conformed to the ‘Declaration of Helsinki for Medical Research involving Human Subjects’. This study was approved by the Ethics Committee of the Chinese PLA General Hospital (Approval No. LL-LCSY-2026-04) before formal data extraction, analysis, and manuscript preparation for this retrospective study.

Inclusion criteria were: (i) age <40 years; (ii) body mass index (BMI) <35 kg/m^2^; (iii) treatment with a follicular-phase, single-type GnRH antagonist protocol; (iv) fresh or frozen embryo transfer in the index cycle; (v) endometrial thickness ≥5 mm on the day of HCG trigger; and (vi) ≤3 previous IVF cycles. Exclusion criteria were: uterine anomalies, hyperprolactinemia, recurrent pregnancy loss, endometriosis, hydrosalpinx, congenital adrenal hyperplasia, thyroid disorders, and chromosomal abnormalities.

The following data were extracted from medical records: patient age; anti-Müllerian hormone(AMH); baseline serum levels of follicle-stimulating hormone (FSH), LH, estradiol (E2), and progesterone (P); starting gonadotropin (Gn) dose; total Gn dose; number of oocytes retrieved; number of normally fertilized (two-pronuclear, 2PN) oocytes; number of embryos obtained; number of embryos transferred; and serum E2, P, FSH, and LH levels on the day of HCG administration.

### Stimulation protocols

In this study, all patients received the GnRH-ant protocol. Recombinant follicle-stimulating hormone (rFSH, Gonal-f, Merck Serono, Darmstadt, Germany) was used on the second or third day of menstrual cycle, and the initial dose was performed according to the individual circumstances, with doses ranging from 112.5 to 375 IU per day. B-ultrasound examination and sex hormone were used to monitor follicular growth to adjust the dose of gonadotropin (Gn). When the follicle mean diameter reached 14 mm or E2 serum levels > 300 pg/ml; GnRH-ant (Cetrotide, Merck, Kenilworth, NJ, USA) was injected from stimulation day 5 or 6 until HCG trigger day. When three follicles reached a mean diameter of 18 mm or two follicles reached a mean diameter of 20 mm, 0.25 mg of recombinant HCG (Ovidrel, Merck Serono S.A., Beijing) was administered subcutaneous injection.

### Embryo transfer and follow-up

Oocyte retrieval was performed by transvaginal ultrasonography guidance 36–38 hours post-HCG administration. Fertilization was accomplished via either conventional IVF or ICSI, based on semen parameters. Embryo transfer was conducted either at the cleavage stage (day 3 post-retrieval) or blastocyst stage (day 5–6 post-retrieval), with a maximum of two embryos transferred. Embryo grading followed standardized morphological criteria (10). In our center, high-quality day-3 embryos were defined as those exhibiting 6–10 blastomeres of relatively uniform size with ≤20% fragmentation. Blastocysts were classified into stages 1–6 according to expansion and hatching status, with those at stages 3–6 undergoing additional evaluation of inner cell mass (ICM) and trophectoderm (TE) quality. Blastocysts graded ≥3BB were considered high-quality. Luteal phase support was initiated on the first day following oocyte retrieval and continued through 10 weeks of gestation. Serum β-HCG levels were measured 14 days post-transfer.

### Outcome measures and definition

Biochemical pregnancy loss: A pregnancy diagnosed only by the detection of HCG in serum or urine without a gestational sac visualized by vaginal ultrasound at the 5th week of pregnancy. Clinical pregnancy was confirmed by transvaginal ultrasound detection of an intrauterine gestational sac with fetal cardiac activity (11). Early pregnancy loss was defined as spontaneous demise of a clinical pregnancy prior to 12 weeks of gestation. Live birth was documented as the delivery of at least one viable neonate beyond 28 weeks of gestation per initiated embryo transfer cycle. Ectopic pregnancy was diagnosed when implantation occurred outside the uterine cavity, confirmed by ultrasonographic findings, laparoscopic visualization, or histopathological evidence. Preterm birth was defined as delivery occurring between 28 and 37 completed weeks of gestation (12).

### Clinical feature selection, model construction and performance evaluation

Patients with any missing data were excluded. The final study cohort was randomly divided into a training set and an internal validation set at a ratio of 7:3. All baseline characteristics were comparable between the two sets (all *P* > 0.05), as summarized in **Table 1**. Patients without outcome data or with incomplete key clinical information required for endpoint definition were excluded from the analysis. Candidate variables with more than 20% missing values were excluded from model development to reduce uncertainty associated with excessive imputation. For the remaining variables with missingness ≤20%, missing values were handled using multiple imputation. The number and percentage of missing values for each candidate variable, as well as the corresponding handling method, are summarized in **Supplementary Table 1**. All subsequent variable selection and model development steps were performed exclusively within the training set.

**Table 1:** Comparison of baseline characteristics between the training and validation cohorts.

Characters	Training set (n=399)	Validation set (n=171)	Entire set (n=570)	P value
Age (years)	32 (23-38)	33 (23-38)	33 (23-38)	0.490
AMH (ng/ml)	3.39 (0.01-30.11)	3.52 (0.01-18.26)	3.44 (0.01-30.11)	0.997
AFC	16 (1-29)	16 (2-29)	16 (1-29)	0.950
BMI	22.1 (16.36-35.00)	22.04 (16.02-33.13)	22.04 (16.02-35.00)	0.951
Basal LH (mIU/ml)	4.57 (0.20-39.83)	4.43 (1.07-20.72)	4.49 (0.20-39.83)	0.939
Basal FSH (mIU/ml)	6.68 (2.11-48.1)	6.50 (2.99-25.35)	6.61 (2.11-45.99)	0.995
Basal E2 (pg/ml)	5.19 (0.97-8.14)	5.14 (3.51-7.93)	5.18 (0.97-8.14)	0.833
Basal PRL (ng/ml))	3.87 (0.00-9.52)	3.96 (0.00-7.04)	3.90 (0.00-9.52)	0.360
Basal T (ng/dl)	0.38 (0.00-2.10)	0.37 (0.00-1.85)	0.38 (0.00-2.1)	0.640
Basal P (ng/ml)	0.48 (0.00-8.14)	0.50 (0.05-3.36)	0.49 (0.00-8.14)	0.414
HCG-LH	2.66 (0.27-30.51)	2.42 (0.40-28.80)	2.62 (0.27-30.51)	0.720
HCG-E2	11.51 (0.75-14.01)	11.46 (2.46-13.71)	11.51 (0.75-14.01)	0.534
HCG-P	1.17 (0.06-19.40)	1.25 (0.12-8.20)	1.20 (0.06-19.40)	0.334
Gn-dose (IU)	7.82 (4.13-9.40)	7.82 (4.13-8.56)	7.82 (4.13-9.40)	0.998
Gn duration (days)	10 (3-16)	10 (3-16)	10 (3-16)	0.832
Antagonist-dose (IU)	7.50 (1.25-27.50)	7.50 (1.25-27.50)	7.50 (1.25-27.50)	0.311
Antagonist-days	4 (1-18)	4 (1-11)	4 (1-18)	0.253
Endometrial-thickness (mm)	10.1 (4.0-19.2)	10.0 (3.0-17.0)	10.0 (3.0-19.2)	0.116
Endometrial-morphology				0.990
A	103	42	145	
B	254	113	367	
C	42	16	58	
Fertilization way				0.625
IVF	244	112	356	
ICSI	155	59	214	

AMH, anti-Müllerian hormone; AFC, antral follicle count; BMI, body mass index; LH, luteinizing hormone; FSH, follicle-stimulating hormone; E2, estradiol; PRL, prolactin; T, testosterone; P, progesterone; HCG, human chorionic gonadotropin; Gn, gonadotropin; IVF, *in vitro* fertilization; ICSI, intracytoplasmic sperm injection.

Continuous variables are presented as median (range), and categorical variables are presented as numbers.

A total of 21 candidate variables were collected as candidate predictors. Variable selection was performed separately for three binary outcomes: (i) available embryo (at least one transferable embryo), (ii) pregnancy (clinical pregnancy after embryo transfer), and (iii) delivery (live birth). For each outcome, a two-stage selection procedure was implemented. In the first stage, the Boruta algorithm, a random forest-based wrapper method, was applied to identify all variables significantly associated with the outcome compared with shadow attributes. Variables confirmed as important by Boruta were advanced to the second stage, where least absolute shrinkage and selection operator (LASSO) regression was performed (13, 14). The optimal penalty parameter (λ) was determined via 10-fold cross-validation using the one-standard-error rule. Variables with non-zero coefficients in the final LASSO model were retained as predictors for the respective outcome. All analyses were conducted in R using the “Boruta” and “glmnet” packages.

Comparison of the final predictor sets across the three outcomes revealed that the variables selected for pregnancy overlapped most extensively with those selected for available embryo and delivery. Consequently, pregnancy was adopted as the primary endpoint for model construction. In the training set, a multivariable logistic regression model was fitted using the predictors identified for pregnancy, and a nomogram was generated to enable individualized prediction of clinical pregnancy probability.

Model performance was assessed in the training cohort and the internal split-sample validation cohort. Calibration was examined via calibration plots (predicted versus observed probabilities). Discrimination was evaluated by the area under the receiver operating characteristic curve (AUC) with 95% confidence intervals estimated using the DeLong method. The nomogram, receiver operating characteristic curves, and calibration plots were produced using the “rms”, “pROC”, and “ggplot2” packages (15). Decision curve analysis (DCA) was performed to evaluate the clinical utility of the prediction model by quantifying the net benefit across a range of threshold probabilities (16).

### Statistical analysis

Normality of continuous variables was assessed using the Shapiro-Wilk test. As all continuous variables significantly deviated from a normal distribution (all *P* < 0.05), they are presented as median with range (minimum-maximum), and comparisons between groups were performed using the Kruskal-Wallis test. Categorical variables are summarized as frequencies and percentages and were compared using Fisher’s exact test. Calibration was assessed using calibration curves, calibration intercept, and calibration slope. An ideal calibration intercept is 0, and an ideal calibration slope is 1. Bootstrap internal validation with 1,000 resamples was performed to assess model optimism, and optimism-corrected performance measures were reported. Data processing, variable selection, modeling, and visualization were performed in R software (version 4.2.0).

## Results

### Baseline characteristics of study cohorts

**Figure 1:**
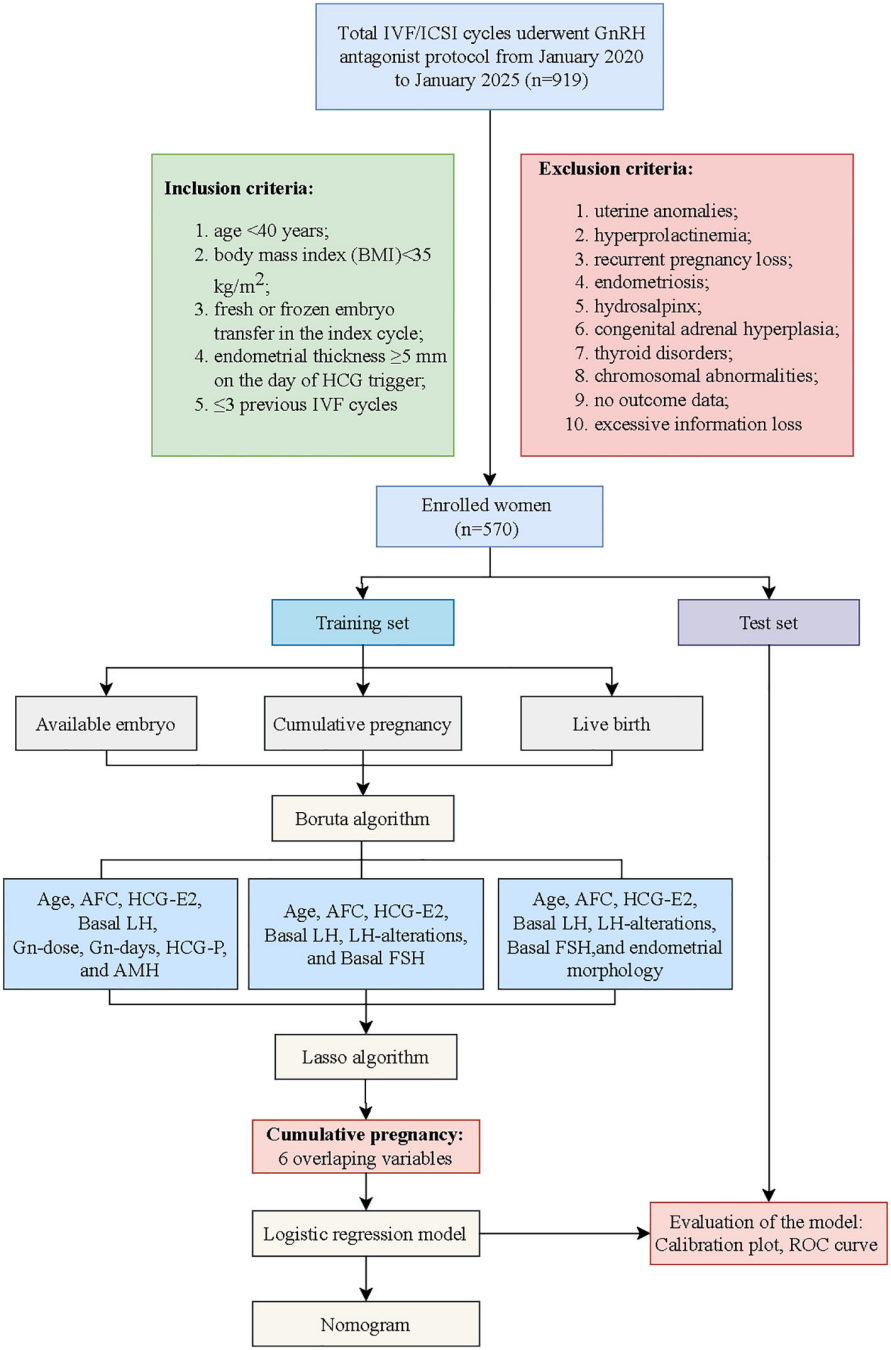
A flowchart depicting the study population and design. The flowchart shows patient screening, cohort allocation, feature selection using the Boruta and LASSO algorithms, model construction, and model evaluation. IVF, *in vitro* fertilization; ICSI, intracytoplasmic sperm injection; GnRH, gonadotropin-releasing hormone; BMI, body mass index; HCG, human chorionic gonadotropin; AFC, antral follicle count; AMH, anti-Müllerian hormone; LH, luteinizing hormone; FSH, follicle-stimulating hormone; HCG-E2, serum estradiol level on the day of HCG trigger; HCG-P, serum progesterone level on the day of HCG trigger; Gn-dose, total gonadotropin dose; Gn-days, duration of gonadotropin stimulation; ROC, receiver operating characteristic; LASSO, least absolute shrinkage and selection operator.

The methodology employed in this study is illustrated by the flowchart shown in **Figure 1**. From January 2020 to January 2025, 919 female patients underwent controlled ovarian stimulation using a GnRH antagonist protocol in our center. After screening according to the predefined inclusion and exclusion criteria, 605 patients were initially eligible. Among them, 17 patients without outcome data and 18 patients with incomplete key clinical information were excluded, resulting in a final analytical cohort of 570 patients. The baseline and characteristics of the overall cohort are summarized in **Table 1**. The median age of the enrolled women was 33 years (interquartile range [IQR], 30-35), with a median body mass index (BMI) of 22.04 kg/m^2^ (IQR, 20.17-24.61). Primary infertility was reported in 48.86% of the participants. Ovarian reserve parameters included a median AMH level of 3.44 ng/mL (IQR, 1.85-5.30) and a median antral follicle count (AFC) of 16 (IQR, 10-19). Clinical pregnancy occurred in 218 of 570 patients overall (38.2%), including 142 of 399 patients in the training cohort (35.6%) and 76 of 171 patients in the validation cohort (44.4%). With six predictors included in the final model, the events-per-variable ratio in the training cohort was 23.7, exceeding the commonly recommended minimum threshold of 10.

### Feature selection for clinical model

**Figure 2:**
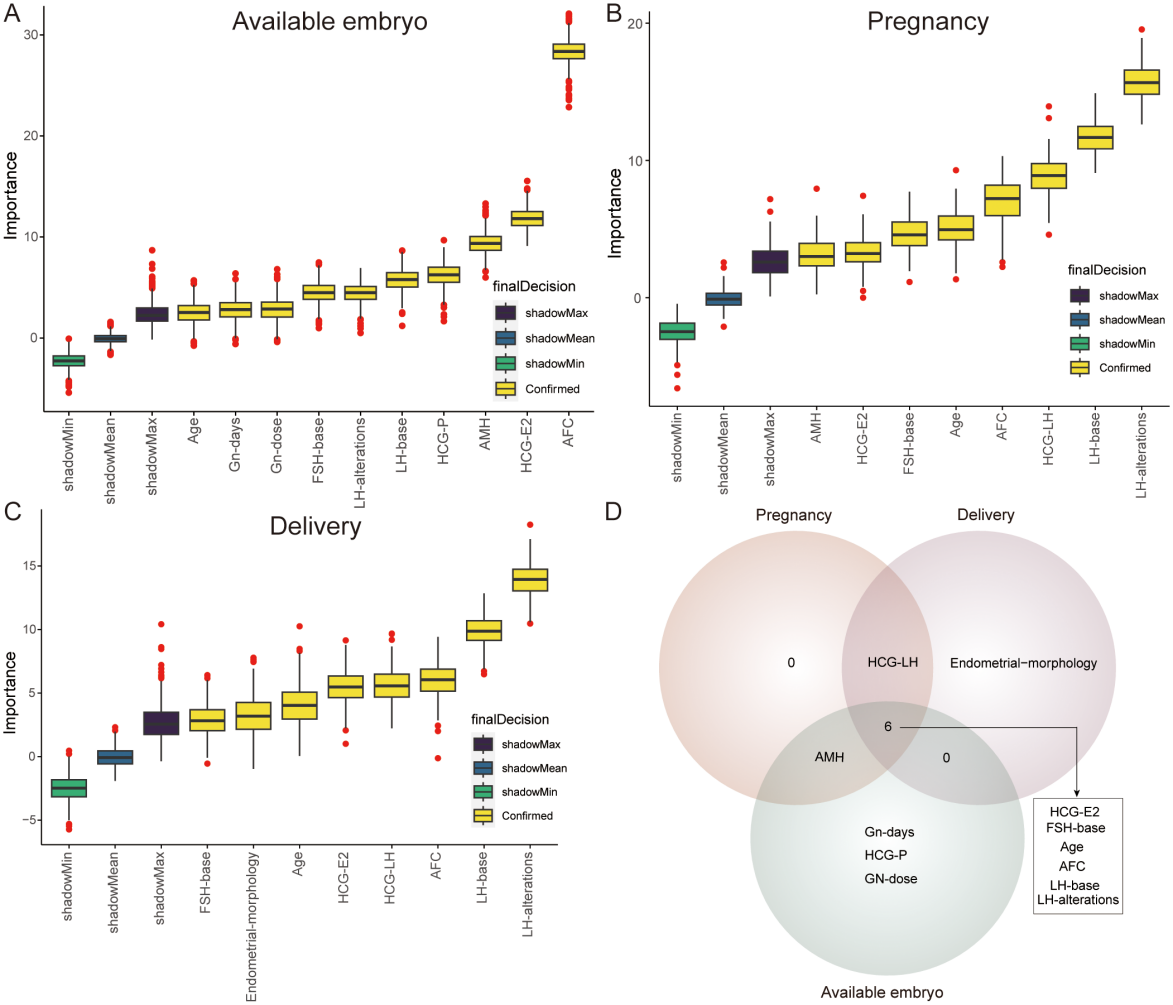
Variable selection using the Boruta algorithm across three IVF outcomes. **(A–C)** Boruta importance plots for **(A)** available embryo, **(B)** pregnancy, and **(C)** delivery, respectively. **(D)** Venn diagram illustrating the overlap of confirmed variables among the three outcomes. AFC, antral follicle count; AMH, anti-Müllerian hormone; FSH-base, basal follicle-stimulating hormone level on menstrual cycle day 2 or 3; LH-base, basal luteinizing hormone level on menstrual cycle day 2 or 3; HCG-E2, serum estradiol level on the day of human chorionic gonadotropin trigger; HCG-LH, serum luteinizing hormone level on the day of human chorionic gonadotropin trigger; HCG-P, serum progesterone level on the day of human chorionic gonadotropin trigger; LH alterations, the change in luteinizing hormone level between baseline and the day of HCG trigger; Gn-dose, total gonadotropin dose; Gn-days, duration of gonadotropin stimulation.

Among the initial candidate variables, six variables with >20% missing values were excluded (CA125, smoking history, occupation, years of infertility, erythrocyte sedimentation rate (ESR) and insulin resistance), and the remaining missing values in retained variables were handled using multiple imputation. The missing rate and handling method for each candidate variable are summarized in **Supplementary Table 1**. Additionally, we introduced the difference in LH levels between the baseline day and the day of HCG trigger as a separate variable, termed “LH-alterations”, to better evaluate the impact of LH changes on assisted reproductive outcomes. LH alterations were calculated as the directional difference between LH level on the day of HCG trigger and basal LH level on menstrual cycle day 2 or 3: LH alterations = LH on HCG trigger day − basal LH. A negative value indicates a decrease in LH during controlled ovarian hyperstimulation, whereas a positive value indicates an increase. After excluding variables with significant collinearity, 21 variables were finally taken into the process of feature selection. The dataset was randomly partitioned into training and testing subsets at a ratio of 7:3. All baseline characteristics were comparable between the training and internal validation cohort (all *P* > 0.05). To identify the most significant predictors correlated with the three clinical outcomes (the number of transferable embryos, cumulative pregnancies, and live births), the Boruta algorithm was employed. **Figures 2A–C** illustrate the distribution of importance scores for all candidate variables associated with each outcome, using shadow features as a baseline to evaluate the significance of true predictors. As shown in **Figure 2A**, nine variables were confirmed as crucial features for the available embryo outcome (represented by yellow boxplots). Ranked by median importance scores from highest to lowest, AFC demonstrated the highest value, followed by HCG-E2, AMH, HCG-P, LH-alterations, FSH-base, Gn-dose, Gn-days, and Age. Although variables such as Age and Gn-days exhibited relatively lower importance scores, all confirmed features exceeded the maximum importance of the shadow variables. Regarding the pregnancy outcome (**Figure 2B**), eight key predictive factors were confirmed: AMH, HCG-E2, FSH-base, Age, AFC, HCG-LH, LH-base, and LH-alterations. Among these features, LH-alterations and LH-base displayed the highest importance scores, whereas AMH and HCG-E2 showed relatively lower but still significant importance compared to the shadow variables. For the delivery outcome (**Figure 2C**), the Boruta algorithm similarly identified eight key features, including Endometrial-morphology, FSH-base, Age, HCG-E2, HCG-LH, AFC, LH-base, and LH-alterations. Consistent with the pregnancy outcome, LH-alterations and LH-base again ranked as the most important variables for predicting delivery. To further explore the overlapping and outcome-specific relationships among these predictive factors, we constructed a Venn diagram illustrating the intersections of the confirmed variable sets across the three outcomes (**Figure 2D**). The Venn diagram revealed that 6 variables served as common predictive features shared by all three outcomes: Age, AFC, HCG-E2, Basal LH, LH-alterations, and Basal FSH. Multicollinearity was reassessed among the six final predictors. Pairwise Spearman correlation coefficients were all below 0.6, and all VIF values were below 5, indicating no substantial multicollinearity in the final model.

**Figure 3:**
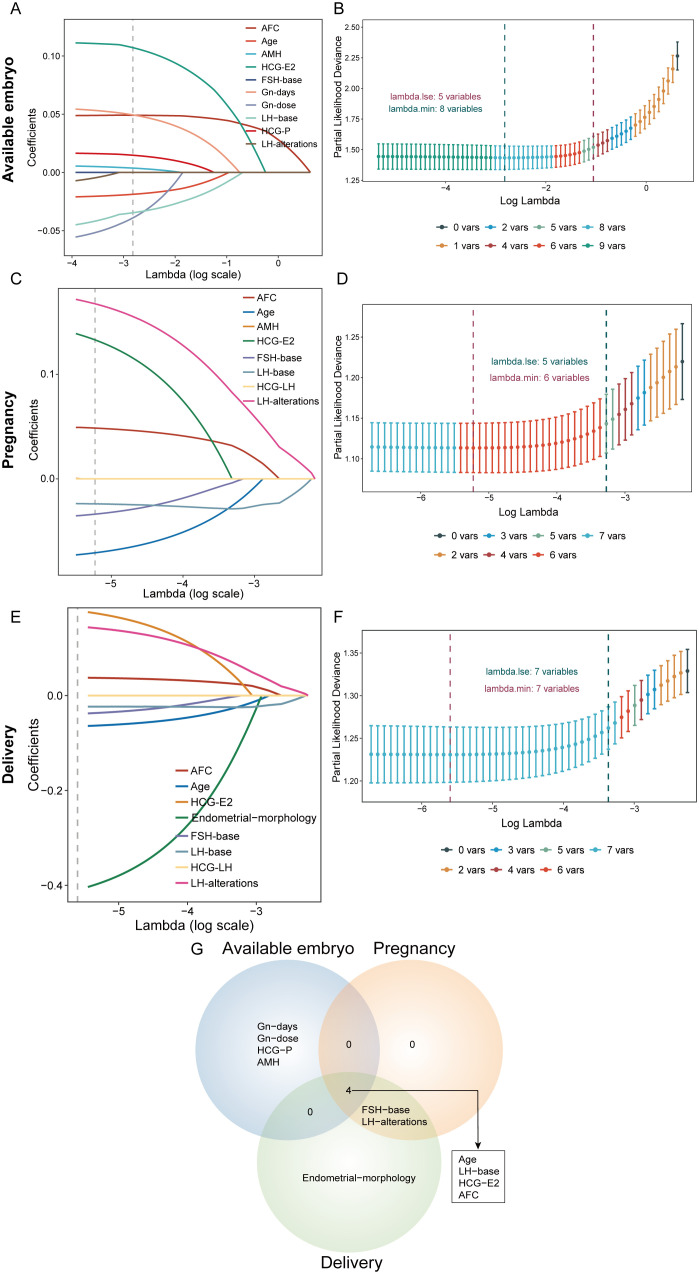
Identification of key predictors using the LASSO algorithm for three IVF outcomes. **(A–F)** LASSO logistic regression results for **(A, B)** available embryo, **(C, D)** pregnancy, and **(E, F)** delivery, respectively. **(G)** Venn diagram illustrating the overlap of LASSO-selected predictors among the three outcomes. AFC, antral follicle count; AMH, anti-Müllerian hormone; FSH-base, basal follicle-stimulating hormone level on menstrual cycle day 2 or 3; LH-base, basal luteinizing hormone level on menstrual cycle day 2 or 3; HCG-E2, serum estradiol level on the day of human chorionic gonadotropin trigger; HCG-LH, serum luteinizing hormone level on the day of human chorionic gonadotropin trigger; HCG-P, serum progesterone level on the day of human chorionic gonadotropin trigger; LH alterations, the change in luteinizing hormone level between baseline and the day of HCG trigger; Gn-dose, total gonadotropin dose; Gn-days, duration of gonadotropin stimulation; LASSO, least absolute shrinkage and selection operator.

To further refine the predictor set and identify the most clinically relevant variables for the three reproductive outcomes, LASSO logistic regression was employed (**Figures 3A–F**). The coefficient path plots (**Figures 3A, C, E**) illustrate the shrinkage of coefficients for each variable as the lambda penalty increases, while the partial likelihood deviance plots (**Figures 3B, D, F**) show the cross-validation error to determine the optimal tuning parameter. For the available embryo outcome (**Figures 3A, B**), the LASSO model selected a set of predictors at the optimal lambda value. The selected variables included AFC, Age, AMH, HCG-E2, FSH-base, Gn-days, Gn-dose, HCG-P, and LH-alterations, as indicated by the non-zero coefficients observed in the path plot (**Figure 3A**). For the pregnancy outcome (**Figures 3C, D**), the model convergence led to the retention of eight key predictors at the optimal lambda, including AFC, Age, AMH, HCG-E2, FSH-base, LH-base, HCG-LH, and LH-alterations, with LH-alterations displaying the highest coefficients. For the delivery outcome (**Figures 3E, F**), the LASSO algorithm identified AFC, Age, HCG-E2, Endometrial-morphology, FSH-base, LH-base, HCG-LH, and LH-alterations as the candidate predictive features. To visualize the specific predictive features retained in the final models and their relationship across outcomes, a Venn diagram was constructed (**Figure 3G**). Four variables: Age, LH-base, HCG-E2, and AFC, were identified as common independent predictors for available embryo, pregnancy, and delivery. A distinct set of two variables, FSH-base and LH-alterations, overlapped between available embryo and pregnancy. For delivery outcomes, endometrial morphology was identified as another confirmed features, while for available embryos, stimulation duration of Gn-dose, Gn-days, HCG-P, and AMH were also determined to be confirmed features. Variables selected only for other outcomes were not included in the final model but are shown in the revised **Figure 3G** for transparency. After Boruta and LASSO selection were performed for each outcome, we compared the retained predictors across available embryo, clinical pregnancy, and live birth. Variables that were repeatedly selected across outcomes and had clear clinical relevance were prioritized for the final model. Clinical pregnancy was selected as the primary endpoint because live birth events were limited, with some patients still under follow-up. Therefore, age, AFC, HCG-E2, basal LH, LH alterations, and basal FSH were retained to construct a parsimonious and clinically interpretable model for clinical pregnancy prediction.

### Evaluation of clinical model

**Figure 4:**
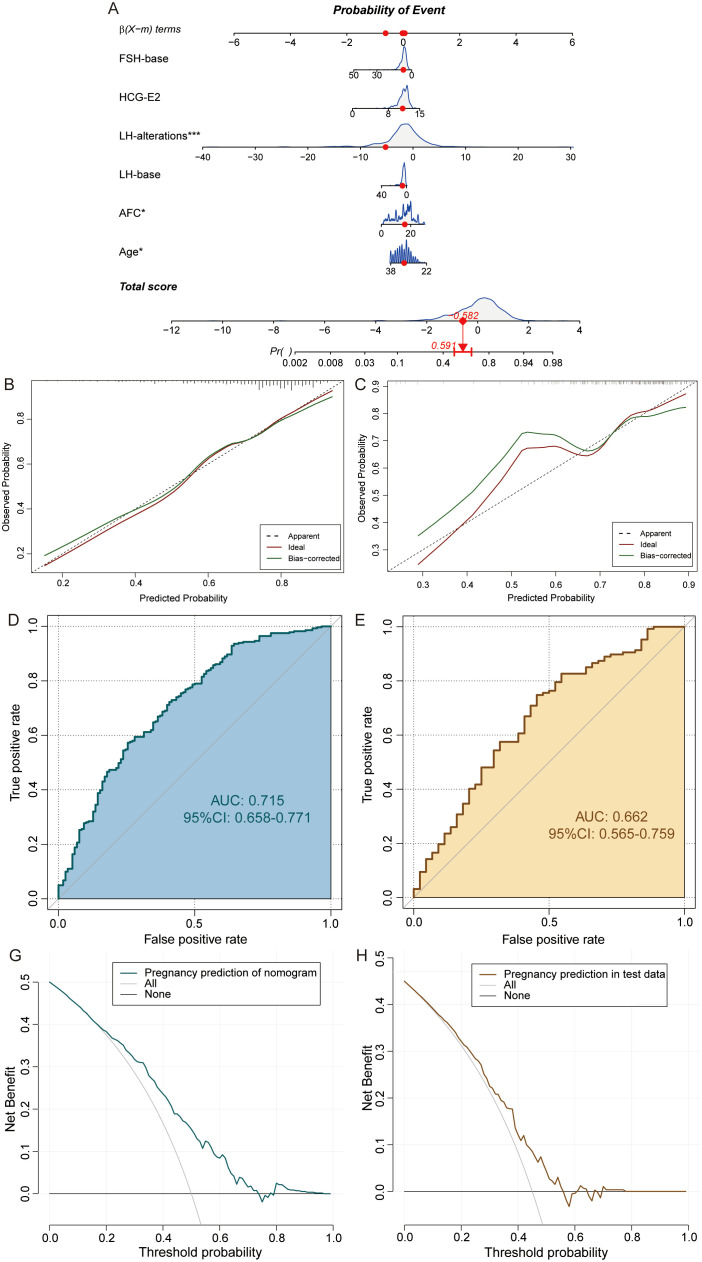
Nomogram for predicting clinical pregnancy and assessment of model performance. **(A)** Nomogram constructed from the multivariable logistic regression model developed in the training set. **(B, C)** Calibration plot comparing the predicted probabilities of clinical pregnancy with the observed proportions in the **(B)** training and **(C)** validation set. **(D, E)** Receiver operating characteristic (ROC) curve for the prediction model in the **(D)** training and **(E)** validation set. **(G, H)** Decision curve analysis of the nomogram in the training cohort and validation cohort. AFC, antral follicle count; FSH-base, basal follicle-stimulating hormone level on menstrual cycle day 2 or 3; LH-base, basal luteinizing hormone level on menstrual cycle day 2 or 3; HCG-E2, serum estradiol level on the day of human chorionic gonadotropin trigger; LH alterations, the change in luteinizing hormone level between baseline and the day of HCG trigger; AUC, area under the receiver operating characteristic curve; CI, confidence interval; ROC, receiver operating characteristic; DCA, decision curve analysis. In panel (A), * p < 0.05, *** p < 0.001.

In this study, we developed and validated a novel, clinician-friendly nomogram that quantifies probability of clinical outcomes for patients undergoing IVF. **Figure 4A** presents the predictive nomogram for clinical pregnancy outcomes following embryo transplantation, which integrates the six clinical confirmed features into a single graphical tool. The nomogram demonstrated moderate discriminative ability in the training cohort, and limited discriminative ability in the validation cohort. The calibration curve for the validation cohort (**Figures 4B, C**) showed close alignment between the predicted probabilities of clinical pregnancy and the observed outcomes across the entire risk spectrum. In the derivation cohort, the area under the receiver operating characteristic curve (AUC) was 0.715 (95% CI, 0.658-0.771). This performance was consistently high in the internal validation cohort using bootstrapping (AUC, 0.662; 95% CI, 0.565-0.759). The ROC curves for both cohorts are presented in **Figures 4D**, **E**. DCA was performed to further assess the potential clinical utility of the nomogram (**Figures 4G, H**). In the training cohort, the model showed a consistently higher net benefit than the treat-all and treat-none strategies across a clinically relevant threshold probability range of approximately 0.20 to 0.70, indicating potential usefulness for risk stratification. In the validation cohort, the model also provided a higher net benefit than the two reference strategies within a lower-to-moderate threshold probability range, mainly below approximately 0.50. Calibration was further quantified using calibration intercept and calibration slope (**Supplementary Figure 1**). In the training cohort, the calibration intercept was -0.264 and the calibration slope was 1.040. In the validation cohort, the calibration intercept was 0.156 and the calibration slope was 0.980. Bootstrap internal validation with 1,000 resamples showed a bootstrap-corrected calibration slope of 0.8766 and an optimism-corrected C-index of approximately 0.692, suggesting acceptable calibration but modest discriminative performance.

## Discussion

This study aimed to develop and validate a clinical prediction model for the GnRH-antagonist ovarian stimulation protocol, identifying six key determinants of the clinical pregnancy outcome: Age, AFC, HCG-E2, LH-alterations, Basal FSH and LH. Previous studies have confirmed that some of the above factors have significant impacts on the pregnancy outcomes of assisted reproduction.

AFC and basal FSH are routinely used markers of ovarian reserve in clinical practice and have been associated with the size of the primordial follicle pool and reproductive outcomes (17, 18). Elevated basal FSH has been reported to correlate with poorer pregnancy outcomes, particularly among women of advanced reproductive age (19–22) Similarly, serum estradiol (E2) on the day of HCG trigger has been associated with IVF outcomes, and lower E2 levels may reflect impaired follicular development or reduced follicular activity (23–25). In the present study, these conventional indicators were retained in the final model, supporting their continued relevance in the prediction of clinical pregnancy after GnRH antagonist stimulation.

Beyond these established predictors, the main finding of our study is that dynamic LH alteration between the early follicular phase and the day of HCG trigger may provide additional information beyond a single-point LH measurement. Previous studies have mainly focused on absolute LH levels at specific time points, whereas evidence regarding LH changes during controlled ovarian hyperstimulation remains limited. LH is involved in follicular development, steroidogenesis, and endometrial receptivity (26–28). In GnRH antagonist cycles, basal hormone levels reflect the endogenous endocrine status before antagonist-induced pituitary suppression, whereas hormone levels on the day of HCG trigger represent the late follicular endocrine environment after controlled stimulation and antagonist administration. Therefore, the change in LH between these two time points may capture both the baseline endocrine reserve and the degree of LH suppression during stimulation. From a biological perspective, LH participates in ovarian steroidogenesis through regulation of theca and granulosa cell function. Follicular steroid synthesis involves conversion of cholesterol-derived pregnenolone through the Δ5 and Δ4 pathways, leading to the production of androgen precursors, estrogen, and progesterone-related metabolites (29–33). Previous studies have suggested that LH levels in the late follicular phase may influence estradiol production under GnRH antagonist protocols (34, 35). LH may also affect follicular development through growth factors such as insulin-like growth factor 1 and vascular endothelial growth factor, which are involved in granulosa cell proliferation and differentiation (36). Thus, excessive LH suppression may theoretically impair follicular steroidogenesis, whereas inadequate suppression may increase the risk of premature LH rise or premature luteinization, both of which may negatively affect follicular quality and reproductive outcomes (37, 38). These mechanisms provide a possible biological explanation for why LH alteration, rather than a single LH value alone, may be associated with clinical outcomes. Compared to using recombinant follicle-stimulating hormone (r-FSH) alone, appropriately increasing the LH level yields a greater number of oocytes and higher embryo implantation rates.

In our study, LH alteration was introduced as an independent dynamic variable and was associated with available embryo, clinical pregnancy, and live birth outcomes. This finding suggests that monitoring the direction and magnitude of LH changes during stimulation may help clinicians better understand individual endocrine responses in GnRH antagonist cycles. However, given the retrospective and observational nature of this study, our results should not be interpreted as evidence that intervention based on LH alteration, such as LH supplementation or adjustment of antagonist timing, can improve pregnancy outcomes. Such strategies require confirmation in prospective interventional studies. At present, LH alteration should be considered a candidate dynamic marker that may provide supplementary information for risk stratification rather than a direct basis for treatment modification.

Our predictive model integrated several clinically relevant determinants identified through Boruta and LASSO analyses, including LH alterations, HCG-E2, age, AFC, basal FSH, and basal LH. These variables reflect both conventional ovarian reserve indicators and endocrine changes during controlled ovarian hyperstimulation, thereby providing a multidimensional assessment of factors associated with pregnancy outcomes in GnRH antagonist cycles. Among these predictors, LH alterations showed an important contribution to clinical pregnancy prediction, suggesting that dynamic LH changes between baseline and the day of HCG trigger may provide additional information beyond single-time-point hormone measurements. Nevertheless, the modest validation AUC indicates that LH alterations, even when combined with conventional clinical and ovarian reserve markers, cannot fully capture the complexity of clinical pregnancy after ART. Therefore, the current model should be interpreted as an exploratory risk-stratification tool rather than a definitive decision-making model. Taken together, our findings highlight the potential relevance of dynamic hormonal assessment in GnRH antagonist cycles and may provide supplementary information for individualized risk evaluation in clinical practice.

However, several limitations should be acknowledged. First, this was a retrospective, single-center study based on routinely collected clinical data; therefore, selection bias and center-specific practice patterns cannot be fully excluded. Second, the model was constructed using a limited set of available clinical and hormonal variables. Several potentially important factors, such as embryo quality, endometrial receptivity, laboratory procedures, treatment-year effects, and more detailed dynamic endocrine profiles during controlled ovarian hyperstimulation, were not included, which may partly explain the modest predictive performance. Third, the model showed only moderate discrimination in the training cohort and limited discrimination in the validation cohort, indicating restricted clinical applicability. Thus, although LH alteration may be a potentially informative dynamic marker in GnRH antagonist cycles, the current model should be interpreted with caution and should not be used as a stand-alone tool for clinical decision-making. In addition, fresh and frozen embryo transfer cycles were combined to preserve sample size, because the number of fresh transfer cycles and corresponding pregnancy or live birth events was limited. However, differences in endometrial preparation, hormonal environment, and transfer strategy may have introduced heterogeneity into the model. Future prospective multicenter studies with larger sample sizes are needed to incorporate additional clinically relevant predictors, externally validate the model, and separately evaluate its performance in fresh and frozen embryo transfer cycles.

Most previous studies have primarily focused on the absolute LH level on the day of HCG trigger, with limited attention given to the dynamics of LH fluctuation during stimulation. In contrast, our model demonstrates that changes in LH levels significantly influence IVF outcomes. These findings suggest that clinicians should not only consider the LH value on the trigger day but also take into account basal LH concentrations for a more comprehensive assessment.

## Conclusion

In conclusion, our findings suggest that LH dynamics during controlled ovarian stimulation, rather than the absolute LH value on the HCG trigger day alone, are associated with IVF outcomes in GnRH antagonist cycles. By incorporating LH alterations together with basal LH and other routinely available clinical indicators, this study provides a more comprehensive perspective for understanding individual endocrine responses during ovarian stimulation. These results highlight the potential clinical relevance of monitoring LH changes from baseline to the trigger day and suggest that basal LH levels should not be overlooked in routine assessment. Nevertheless, given the retrospective design of this study, further prospective multicenter studies are warranted to validate these findings and to determine whether LH-based individualized assessment can contribute to improved ART management.

## Data Availability

The original contributions presented in the study are included in the article/**Supplementary Material**. Further inquiries can be directed to the corresponding authors.
