# Supplementary material for: Development and internal validation of a prediction model for clinical pregnancy in GnRH antagonist cycles: a retrospective cohort study
Source: Front Endocrinol (Lausanne). 2026 Jul 28;17:1916298. doi: 10.3389/fendo.2026.1916298 (PMC13457315; doi:10.3389/fendo.2026.1916298)
Supplement: Supplementary file 1 [file Supplementaryfile1.docx]

**Supplementary Figure S1. Quantitative calibration assessment of the prediction model.**
Calibration plots with calibration intercept and slope in the training cohort and validation cohort. The calibration intercept and slope were -0.264 and 1.040 in the training cohort, and 0.156 and 0.980 in the validation cohort, respectively.
