# Supplementary material for: Development and internal validation of a prediction model for clinical pregnancy in GnRH antagonist cycles: a retrospective cohort study
Source: Front Endocrinol (Lausanne). 2026 Jul 28;17:1916298. doi: 10.3389/fendo.2026.1916298 (PMC13457315; doi:10.3389/fendo.2026.1916298)
Supplement: Supplementary file 2 [file Supplementaryfile2.docx]

**Supplementary Table S1 | Missing data summary of candidate variables**

| ***Va*riable** | **Variable type** | **No. of missing values** | **Missing rate (%)** | **Handling method** |
| --- | --- | --- | --- | --- |
| Age | Continuous | 0 | 0.0 | Included |
| BMI | Continuous | 0 | 0.0 | Included |
| AMH | Continuous | 5 | 0.9 | Multiple imputation |
| AFC | Continuous | 0 | 0.0 | Included |
| Basal FSH | Continuous | 0 | 0.0 | Included |
| Basal LH | Continuous | 0 | 0.0 | Included |
| Basal E2 | Continuous | 2 | 0.4 | Multiple imputation |
| hCG-E2 | Continuous | 3 | 0.5 | Multiple imputation |
| hCG-LH | Continuous | 3 | 0.5 | Multiple imputation |
| LH alterations | Continuous | 3 | 0.5 | Calculated after imputation / Included |
| CA125 | Continuous | 168 | 29.5 | Excluded, missing rate >20% |
| Smoking history | Categorical | 245 | 43.0 | Excluded, missing rate >20% |
| Occupation | Categorical | 332 | 58.2 | Excluded, missing rate >20% |
| Years of infertility | Continuous | 119 | 20.9 | Excluded, missing rate >20% |
| ESR | Continuous | 188 | 33.0 | Excluded, missing rate >20% |
| Insulin resistance | Categorical/Continuous | 360 | 63.2 | Excluded, missing rate >20% |

**Abbreviations:** AMH, anti-Müllerian hormone; AFC, antral follicle count; BMI, body mass index; LH, luteinizing hormone; FSH, follicle-stimulating hormone; E2, estradiol; PRL, prolactin; T, testosterone; P, progesterone; HCG, human chorionic gonadotropin; Gn, gonadotropin; IVF, in vitro fertilization; ICSI, intracytoplasmic sperm injection.
